# AI and the need for justification (to the patient)

**DOI:** 10.1007/s10676-024-09754-w

**Published:** 2024-03-04

**Authors:** Anantharaman Muralidharan, Julian Savulescu, G. Owen Schaefer

**Affiliations:** 1 Centre for Biomedical Ethics, Yong Loo Lin School of Medicine, National University of Singapore, Singapore, Singapore; 2 Murdoch Children’s Research Institute, Melbourne, VIC Australia; 3 Oxford Uehiro Centre for Practical Ethics, Faculty of Philosophy, University of Oxford, Oxford, UK

**Keywords:** AI, Justifiability, Justification, Explanation, Transparency, Shared decision-making

## Abstract

This paper argues that one problem that besets black-box AI is that it lacks algorithmic justifiability. We argue that the norm of shared decision making in medical care presupposes that treatment decisions ought to be justifiable to the patient. Medical decisions are justifiable to the patient only if they are compatible with the patient’s values and preferences and the patient is able to see that this is so. Patient-directed justifiability is threatened by black-box AIs because the lack of rationale provided for the decision makes it difficult for patients to ascertain whether there is adequate fit between the decision and the patient’s values. This paper argues that achieving algorithmic transparency does not help patients bridge the gap between their medical decisions and values. We introduce a hypothetical model we call Justifiable AI to illustrate this argument. Justifiable AI aims at modelling normative and evaluative considerations in an explicit way so as to provide a stepping stone for patient and physician to jointly decide on a course of treatment. If our argument succeeds, we should prefer these justifiable models over alternatives if the former are available and aim to develop said models if not.

## Introduction

Consider the following situation.

Chemo: Alice has just been diagnosed with advanced cancer. Doctors give her months to live but Alice wants to make it to her granddaughter’s birth, some six months away. After keying in the details of her case into his computer, her physician, Bob, says the following: “According to our algorithm, which optimises for quality adjusted life-years (QALYs), chemotherapy may not be the best option for you. Instead, the algorithm recommends palliative care. However, we can’t tell you why this is the case. As per our own clinical judgment, chemotherapy seems to be the better option for you. However, studies have shown that this algorithm’s recommendations, on average, result in better medical outcomes than the unaided clinical judgment of physicians.” Bob then proceeds to, on the basis of the algorithm, recommend palliative care for Alice.

To be sure, this is not science fiction. IBM’s Watson for Oncology (Jie et al., 2021) is a model that is used to make recommendations about treatment decisions. Individual treatment regimens are classified as recommended, for consideration or not recommended. What, if anything, is wrong with such black-box AI models? A common criticism against them is that they lack transparency (Robnik-Sikonja and Kononenko 2008; Mittelstadt et al., 2016; Selbst and Barocas 2018; Rudin, 2019; Kemper and Kolkman 2019). That is, the claim is that, on black-box models, we do not know how the algorithm made its decision or why the algorithm made the decision it did. In this paper, we argue that this concern with transparency is, at best, incomplete. As we will argue, one thing that is wrong with such models is that they fail to show that the recommendation satisfies the appropriate normative standards. These standards have both epistemic and axiological dimensions. The epistemic aspect of these standards is largely physician-facing; The physician’s role is to ensure that the decisions fit the case-specific and general medical-theoretic evidence. The axiological aspect of these standards is largely patient-facing. The patient’s role is in assessing whether these decisions fit her values and priorities. The focus of this paper will be on the axiological aspects of these standards.

The strategy for this paper is as follows. In Section "Justifiability to the patient", we argue that existing accounts of the doctor-patient relationship and shared decision-making can ground a requirement that clinical decisions fit patients’ values and that patients understand why this is the case. This, in turn reveals why black-box AIs are inadequate even when we know what values they are optimized for. Section "Interpretable AI, explainable AI and the wrong kind of information" argues, using a hypothetical AI model we call Justifiable AI as an illustration, that efforts to increase algorithmic transparency aim at providing the wrong sort of information. Therefore justifiability is a distinct and important desideratum for AIs. We conclude in Sect. “Conclusion”.

## Justifiability to the patient

In this section, we argue, that medical decisions ought to be justifiable to patients. That is, medical decisions ought to fit the patient’s values and priorities and that patients are morally entitled to understand why the decisions do so.

Before we proceed, it is worth spelling out clearly what we mean by justification. It should be clear that at least three notions of justification might be operative here: epistemic, normative and interpersonal. Epistemic justification is about whether beliefs are formed reliably (Goldman, 2011) or well supported by the evidence (Feldman and Conee 1985). Recent work on algorithmic justification has focused on the issue of whether we are epistemically justified in accepting the claims made by algorithms (Durán and Formanek 2018; Durán, 2023). Our paper takes for granted that the AI may be demonstrably reliable on factual issues. Even after securing such reliability, some issues pertaining to justification remain.

The second type, normative justification, refers broadly to decisions or actions fitting certain normative or evaluative criteria. A familiar example is moral justification where an act is morally justified if and only if its right or good-making features outweigh or defeat its bad or wrong-making features (Raz, 1999). One sense in which we are claiming that medical decisions ought to be justifiable to patients is in this normative sense. A medical decision is justifiable to a patient to the degree that it aligns with the patient’s concerns and values. Sometimes options are limited and none of the available options align with patient values very well^1^. The appropriate option in such cases is the one which aligns best.

The third type of justification, interpersonal justification, refers to the making available of reasons for action or belief to another person. We might be tempted to think that it is simply a matter of providing epistemic justification for the proposition that a given option is normatively justified. However, this might be too reductive. Interpersonal justification, while involving the discursive act of “reason giving” need not involve the provision of new epistemic reasons for any given proposition. “Reason giving” qua discursive act involves providing an account or argument that makes explicit why the considerations that count in favour of an act or belief do count in favour of said act or belief (Goldman, 1997). In many cases, these considerations would have been values that the recipient of justification already accepted or propositions that she already knew. In such cases, arguably, no new additional reasons are provided epistemic or otherwise. This is important as we will be arguing that in addition to ensuring that the recommended treatment fits patient values and priorities, doctors have a duty to illuminate for the patient why the latter’s current values and priorities count in favour of that treatment.

This orientation towards the patient’s values is something we already are committed to on a common understanding of shared decision-making and its importance in the clinical setting. To this end, we can rehearse two considerations. Firstly, what counts as benefiting the patient adequately depends on the patient’s circumstances and priorities (Caplan, 1997; Wilkinson and Savulescu 2018). Secondly, concerns about autonomy and respect for persons also require attending to patients’ priorities and choices (Downie, 1994; Pellegrino, 1994; Emanuel and Emanuel, 1992). Given that justifiability to patients is an important desideratum of medical decisions, it is important for decision support algorithms to be able to aid clinicians in justifying medical decisions.

### Shared decision-making and justifiability to patients

With regards to the first consideration, the exact mixture of outcomes such as longevity and comfort that would most benefit someone can differ from person to person based on their exact circumstances and priorities. Consider, for instance, a case where two people have different priorities.

Cancer: Hal and Sal, who have both been diagnosed with cancer, are equally ill and have three weeks to live. Chemotherapy would be able to extend their lives by six months, but would also cause them to experience significant suffering. Hal has settled all his affairs and has no milestone events coming up. Sal’s daughter is giving birth in six months’ time and she would like to be there for the birth of her daughter.

Plausibly Sal, but not Hal would benefit from chemotherapy because she has different priorities than Hal. Whereas for Hal, the immense suffering caused by chemotherapy would make his remaining life worse, even if longer, than it would have without the chemo, the same is not true for Sal. Sal is made better off despite the suffering because the slight extension of life allows her to attend an important milestone event.

This point can be generalised to other cases involving value pluralism. As long as there are multiple dimensions of patient well-being, the same health outcome can affect the wellbeing of patients in different circumstances, and hence with different priorities, to different degrees (Wilkinson and Savulescu 2018).

Engaging with the patient and deliberating with her is important for two reasons. Firstly, it is instrumental for the doctor and patient to figure out which course of treatment would in fact be best for the patient. Systematically achieving congruence with patient priorities requires shared decision-making (Emanuel and Emanuel, 1992; Sandman and Munthe 2009). In addition, engaging in shared decision-making with the patient increases the likelihood of the patient adhering to the treatment plan. This in turn can improve health outcomes (Deniz et al., 2021). Plausibly, this requires that the patient actually understand why the treatment fits her values. She has to have a grasp of the relation between the fact that the decision fits her values and the reasons for why it does (Hills, 2009; Elgin, 2017). This is because understanding that the course of action is right for her can help motivate adherence to that course of action (Colby, 2002).

With regards to the second consideration, autonomy has been well established as a reason in favour of shared decision making and ensuring a degree of fit between patient’s values and the decision. An autonomous life, after all, is one in which one’s authentic preferences are realised because one chooses a course of action in light of them and, in turn, effectively pursues this course of action (Raz, 1986; Sandman and Munthe 2009; Sandman et al., 2012; Ubel et al., 2018).

Crucially, an important part of patient autonomy is the patient being able to see that the decision fits these values. To see why, consider a case where a treatment is best for the patient even after taking into account her priorities. Yet, if the physician simply made their recommendation without deliberating with the patient, we are inclined to think that physician was being objectionably paternalistic. That is to say, the physician sets themselves up as the one who has decision-making authority over the patient’s body, whereas most contemporary accounts of patient autonomy and the doctor-patient relationship vest that authority with the patient themselves.

One might think that this simply is a matter of there being a duty to ensure that patients have adequate epistemic justification to believe that the decision fits her values (Durán, 2023). However, this would be a mistake. Imagine a variant of the above case where the physician makes their recommendation without deliberating with the patient and tells the patient “Trust me, I have known you for the past thirty years and know what you care about. This option is the one that is best for you”. It turns out that the physician is indeed right. Moreover, given that the patient knows that her physician knows her well, she is justified in believing that he has chosen in a way that fits her preferences. Yet, this still seems objectionably authoritarian. The physician has still set themself up as having decision-making authority over the patient’s body. Hence merely supplying the patient with an epistemic justification for believing that the decision fits her values is not enough. Rather what is needed to avoid authoritarianism is the provision of the kind of justification that would engage with the patient’s deliberative capacities. This requires an account that directly attempts to address how the decision fits with the patient’s values and priorities. When the patient has an account or at least possesses the materials by which she could construct an account linking her values to the decision, she is in a position to understand why the decision fits her values.

As we have just seen, whether we are looking at the patient’s best interests or their autonomy, it is important that medical decisions fit patient’s values and further that the patient be understand why this is the case. There are many different models of shared decision-making, each of which weighs patient autonomy and paternalistic considerations differently (Emanuel and Emanuel, 1992; Sandman and Munthe 2009). This can range from the most paternalistic models wherein the physician considers only the patient’s situation and not her values and makes the decision for her, to ones where the physician provides the relevant information and the patient decides on her own. With the exception of the most paternalistic model of shared decision-making, all other models take the patient’s values and priorities into consideration. Given the benefits of ascertaining and giving weight to patient priorities, even if we cared only for patient well-being and not their autonomy, we should eschew the most paternalistic models of decision-making.

### One problem with black-box models: machine paternalism

If this is right, then one problem with black-box AI is that it threatens to take us back to the objectionably paternalistic model of medical decision-making and the doctor-patient relationship. To see why, it is useful to understand exactly what a black-box model is.

A black-box AI, roughly, is an algorithm whose inner-workings are, in principle, opaque to even the programmer. More precisely, algorithmic opacity is the disposition of an algorithm to “resist knowledge and understanding” (Beisbart, 2021, 11643). The specific way in which these algorithms resist knowledge and understanding is that information about the function that maps inputs to outputs is simply not available (Humphreys, 2009; Durán and Formanek 2018). If the information is neither directly available nor can be fully reconstructed, even an ideal agent would not be able to infer what the algorithm’s output will be from the given inputs.

To elaborate, consider that instead of explicitly encoding a particular model or function that maps inputs to outputs in some tractable way, AI models are trained on a given dataset in which the correct classifications are specified in advance. For instance, an AI might be given a large dataset containing information about diagnoses, patient information, prognoses and correct course of treatment^2^. The question of which course of treatment is correct in any individual instance is the aforementioned classification and its correctness is stipulated by the medical experts involved in training the algorithm. For instance, in the Chemo case, a given course of treatment was considered correct just in case it is the one that maximised QALYs as defined in a certain way. The AI devises its own function to relate these pieces of information to each other. For black-box AIs like neural networks, even if researchers know, in general, how these functions are constructed, the actual working of these functions remains opaque to them (London, 2019). This would be because for such black-box AI, it is, in principle, impossible to determine, given all current datapoints, how the model or function evolves with the addition of any one datapoint. To make a contrastive illustration, with a linear regression, we are, in principle, able to determine how the function that best fits all datapoints will change with the addition of any new datapoint. Having information about this function means that an ideal agent would be able to infer what recommendation or output the algorithm will deliver in response to some hypothetical or actual input. Likewise, for any given output and input pairs, knowing the function makes it possible to for at least an ideal reasoner to work out why the algorithm gave that recommendation.

By contrast, for any given recommendation made by a black-box algorithm, it is, in principle, impossible to determine why the algorithm had that output in that instance (Humphreys, 2009; Durán and Formanek 2018; Beisbart, 2021). This is because no information is provided about how the algorithm weighted the various inputs in generating the output. Instead, all that is known is that the algorithm was trained on a given dataset, that it performs with a certain degree of reliability under certain given conditions and that success, in turn, is defined along certain parameters. The second of these, moreover, is known only if trials are subsequently conducted on a different dataset and the output of the algorithm is independently validated. Crucially, knowing that successful or correct classification is defined in certain ways is compatible with algorithmic opacity because the lack of information about the algorithm prevents us from knowing whether the algorithm necessarily classifies according to those parameters. For instance, in the Chemo case, we can know, after validation, that the recommended treatment will likely maximise QALYs. However, since we do not know what the function mapping inputs to outputs is, we do not know whether the AI model is calculating the QALYs for each possible course of treatment and choosing the one which maximises that quantity. We only know that there is some function that maps diagnoses, symptoms, patient history and other inputs to treatment decisions and it very often turns out to be the case that the treatment decision maximises QALYs, not what that function is. The latter is what makes the algorithm a black-box one. Even when we are able to, with a post-hoc explainer, determine the weight given to various parameters in a given case, slight changes in background conditions can result in the model weighting the parameters radically differently. Black-box algorithms are also claimed to be more reliable than physicians for certain applications (Babic et al., 2021; Durán and Jongsma 2021).

We are now able to see why black-box AI threatens to be objectionably paternalistic: Since no justification has been provided, it is unclear how the various normative considerations have contributed to the decision (Veliz et al., 2021). For instance, consider the Chemo case again. Let us assume that we can reasonably believe that the algorithm’s decision, palliative care, maximises QALYs for Alice. While Alice may prefer to maximise life-years at least until her granddaughter’s birth, it may not be clear how stable this preference is. This preference for life extension over discomfort may only hold on the assumption that chemotherapy would give rise to moderate discomfort. Plausibly, if chemotherapy gave rise to severe discomfort for her, she would not be able to tolerate such pain and hence would prefer palliative care under such circumstances. Moreover, for all we know, the algorithm may have decided that palliative care maximises QALYs because it expected her to experience severe discomfort with chemotherapy for a long time.

Even if Alice’s preference for life extension over comfort is stable despite the likelihood of severe discomfort, the preference would still be conditional on chemotherapy sufficiently extending her life for her to see her granddaughter. It is possible that the algorithm might estimate that even chemotherapy may not extend her life enough for her to see the birth of her granddaughter. Hence, it is possible that despite Bob’s best judgment, the algorithm, even if it was trained to maximise life-years, would still recommend palliative care for Alice.

To be clear, these are just possibilities. It could be the case that, as per Bob’s unaided judgment, chemotherapy best fits Alice’s values. What the above considerations do make clear is that knowing what the black-box AI was trained to optimise does not tell us anything about whether Bob should defer to its decision. If Bob were to simply ignore it, the AI would be useless. If Bob were to simply defer to it when there is a match between what the AI was trained to optimize and the patient’s values or, instead, do the opposite whenever there was a mismatch, he would still risk acting paternalistically. Importantly, even if the decision fits the patient’s values, the black-box AI does not help patients learn that it does. Its optimising goals must be articulated and interrogated. Moreover, since no justification is provided for the recommendation, there cannot be a reasoned assessment of the AI output except on the basis of what the physician already knows. But this is just tantamount to ignoring the AI!

The problem with ignoring the AI is that the physician and patient are failing to avail themselves of a tool which would have been useful if it had been useable. Such a tool would, in this context, have aided physicians in meeting their obligations to their patients. This should help underscore why the uselessness of black-box AI in this respect is of moral concern.

The foregoing arguments might suggest that all we need to do is incorporate the patient’s values into the algorithm as Value-flexible AI does (McDougall, 2019). Value-flexible AI works by explicitly eliciting preferences (Ruland & Bakken, 2002) over possible treatment outcomes from the patient and then generating a decision that best fits those preferences. Thus, a part of the parameters of the algorithm includes facts about the patient’s preferences over treatment outcomes. We might imagine that a given black-box algorithm is trained on data that includes information about patients’ preferences over treatment outcomes. When the algorithm encounters a new case, information about the patient’s current preferences is fed into the algorithm in order for it to generate a decision. Yet, at least one worry remains, namely that it is hard to programme in milestone events.

Value-flexibility, while important, is not sufficient for justifiability because it is doubtful that the different dimensions of treatment outcomes and value weightings can be adequately parameterised in all cases. With reference to the Chemo case, it can be hard to capture every possible state of affairs which would affect Alice’s preference ordering over treatment options. What can close this gap is an explicit justification for any given treatment decision which accounts for how these different values are weighed against each other in deciding one way or the other.

Defenders of Value-flexibility might say that another way to close this gap is to be more thorough in accounting for the different dimensions of treatment outcomes and the different potential values at stake. However, this leaves us with a dilemma: On the one hand, in order to capture every possible nuance in a patient’s set of values and principles, the patient would have to fill in a form or survey about her preferences which is so extensive as to make medical decision-making impractical or infeasible. It also doesn’t seem feasible to tailor every AI to every set of values. This form-filling can make visits to the doctor unpleasant in ways that are often underappreciated. Moreover, such information may rapidly change as the patient may initially not want 6 more months of suffering but at the last moment her daughter discovers that she is pregnant. On the other hand, any attempt to standardize such values in order to make the process more efficient will fail to cover individual variation in values and preferences. Even in relatively more homogeneous communities there will always be dissenters from the norm and those who reject even the basic ethical framework that others take for granted. That, is, the degree of personalisation of AI that complete value flexibility requires is neither feasible, nor entirely desirable. Hence, without an explicit justification for the decision, the patient may not know whether the AI’s recommendation fits her values.

Summing up, black-box models, even when augmented with a feasible level of value-flexibility, are not adequate in helping patients figure out which options fit their values. If physicians reflexively accept or reject the AI suggestion, they act in an objectionably paternalistic way.

One might instead suppose that we should not use AI to make such value laden decisions and instead use AIs to determine the medical facts such as they might be. In response to this, there are at least two things we might say. Firstly, IBM’s Watson for Oncology is already being used to make such value-laden decisions Watson will assign various chemotherapy regimens to one of three categories: recommended, for consideration, or not recommended (Jie et al., 2021). Since different chemotherapy regimens may have different trade-offs between longevity and comfort, these decisions would be value-laden as well. This is unlikely to stop and other such value-laden decision-making AI may eventually be developed to make such decisions whether in the medical or non-medical context. This paper examines what is at stake in using such models and argues for algorithmic justifiability as a constraint on models that are to be used for such purposes.

Secondly, it is not clear that the problem of justifiability goes away even if we were to stick to using AI to determine the medical facts. While this paper is focused on justifiability to patients within the clinical context, this is not the only context in which questions of justifiability arise. Questions of algorithmic justifiability arise whenever (a) there are, broadly speaking, substantive standards^3^ that AI outputs ought to satisfy and (b) it is desirable that AI users understand why the output satisfies those constraints. In the context of this paper, one substantive requirement is congruence with patient values and the desirability of understanding why this requirement is satisfied is grounded in certain moral obligations to the patient. The question of exactly what those requirements are and how desirable it is to understand why those requirements are met when AI is used only to determine the medical facts is beyond the scope of the current paper. However, it is not implausible to think that such requirements do exist and, given current practices where physicians provide medical justifications, that it is desirable for someone or other to understand why those requirements are met. Given that requirements of justifiability cannot be waived away, *mutatis mutandis*, the only other option is to ignore the black-box AI’s recommendation, but doing so makes the black-box AI useless.

## Interpretable AI, explainable AI and the wrong kind of information

The above discussion might suggest that the solution to the problem of paternalism in black-box AI is greater transparency about the “reasoning” of the algorithm. However, this would be mistaken. Our argument against the primacy of transparency consists of two claims. Firstly, as we covered in the previous section, it is the compatibility of an AI’s recommendation with the patient’s values and the patient’s understanding of why this is that actually matters since this is ultimately the goal of patient-centric care. And secondly, there is a distinction between the question of whether an algorithm actually weighed the patient’s values and the facts of the case in the right way in coming to a recommendation and whether the recommendation has the right sort of fit with respect to the patient’s values and said facts. This distinction matters because attempts at transparency aim at providing the wrong kind of information. This means algorithmic transparency is neither necessary nor necessarily sufficient to secure justification.

As a first pass on our criticism of transparency, algorithmic transparency is about revealing the way in which an algorithm’s outputs depend on its inputs (Durán, 2021). By contrast, justification, at least in the clinical context, is about making apparent why the decision meets certain normative standards. As we argued in Secttion "Justifiability to the patient", there is a moral requirement for medical decisions to satisfy certain evaluative criteria and for patients to be helped understand why this satisfaction obtains. It is the requirement to satisfy these criteria that is the normative standard that has to be met. Since the algorithmic transparency and justification can and often do come apart, attempts at the former are not often conducive to making justifiable decisions. As we will argue, how the AI *actually* arrived at a decision is not the most important issue for algorithmic justifiability. What matters is that there is a justification accessible to the patient (i.e., which they can evaluate in light of their values, priorities and the facts of their situation) for the AI’s recommendation.

At this point, some might claim that transparency is one way of presenting or arriving at a justification (Durán, 2023). This claim is made initially plausible on the grounds that part of what it means to identify how an algorithm or person came to a decision is to identify which considerations mattered, how much they mattered and in which way these considerations contributed to the decision. These considerations may, in turn, be regarded as the agent’s or algorithm’s reasons for the decision. Moreover, if these reasons are sound, that is, if they are considerations that the patient could accept as meaningful on the basis of her values and which do count sufficiently in favour of the decision, she would have been provided with a justification. However, this may not always be the case and oftentimes it may be that there are sound reasons for the decision which the algorithm did not employ in reaching that decision. Because of this, it is sufficient that patients are provided a *valid* chain of reasoning through which a given recommendation could be arrived. The algorithm need not actually have used that chain of reasoning.

In this case, we want to distinguish between knowing how the algorithm came to its decision or how it comes to decisions in general and understanding why a particular decision fits the patient’s values and priorities. The question of “why” is central to justification. When we are asked to justify a belief, we are being asked why we or, for that matter, anyone in our epistemic situation should (or may) form or maintain that belief. Likewise, when asked to justify a decision, we are being asked why that decision is the right one. In the context of the Chemo case, if the doctor were to recommend palliative care, he would be asked why palliative care is right for the patient. Moreover, understanding why a particular decision fits the patient’s values is what is required by norms of shared decision-making. However, transparency only delivers information about how the algorithm came to its decision. Moreover, the two coincide only when the algorithm happens to weigh the various normative considerations in exactly the same way that the patient does (or at least the way the patient ought to)^4^. However, this coincidence rarely happens even when the decision is ostensibly correct. This is because there are potentially infinitely many “bad” chains of reasoning that could be followed to reach the right decision. These chains of reasoning may count as bad because they involve values and priorities that the patient does not accept, because they involve morally objectionable considerations or because they involve epistemically illicit inferences. As we shall see, the mere fact that the algorithm happened to use a “bad” chain of reasoning does not mean that there is not some “good” chain of reasoning available that, if followed, will lead to the same decision. Since the existence of such a “good” chain of reasoning and the patient’s awareness of its existence is what makes said decision justifiable to the patient, transparency is not often conducive towards justification. To illustrate this argument, we shall specify what success in achieving algorithmic transparency looks like, namely interpretable AI and explainable AI. We will then contrast these types of AI with a hypothetical AI model we call Justifiable AI and show why, transparent AI does not provide the right kind of information required for justification.

### Interpretable AI

Interpretability is about trying to answer the question of “how does this algorithm make decisions?”^5^. In this sense, it is a strong interpretation of a putative transparency requirement. We will use the term interpretable AI to refer to white-box models wherein the answer to this question can be read off from the algorithm’s structure itself. Examples of such models include linear equations with additive weights for each variable, logistic equations and (relatively simple) decision trees. Defenders of the black-box models claim that the price for knowing exactly how the algorithm reaches its output is that it is significantly less reliable than black-box AI (Durán and Jongsma 2021; Babic et al., 2021). Supposedly, this is because simpler models are less able to capture more complex causal relationships and reasoning. However, this claim is disputed (Rudin, 2019).

In any case, interpretable AI achieves global transparency. Every part of the algorithm can be scrutinized. However, being able to see how the algorithm will function in other, hypothetical, and perhaps very different situations does not provide the users with any justification for accepting the decision made by the algorithm in the situation they find themselves in. In fact, as we will see when we compare explainable and justifiable models, even information about how that particular decision was arrived at by the algorithm does not aid in justification, except by sheer coincidence. Given that neither information about how the algorithm functions in coming to a decision in the actual circumstances nor information about how the algorithm functions in other circumstances is necessarily helpful in justifying the output to the user, interpretable AIs are likely unhelpful in coming to a justified decision about whether to accept or reject the algorithm’s recommendation.

### Explainable AI

Explainability in AI is understood as attempting to answer the question of “How did the AI make *this* decision?^6^” It involves tracing back the path-dependence of a specific output on its inputs (Ribeiro et al., 2016; Durán, 2021). We shall use the term explainable AI, to designate a certain kind of post-hoc model. Such models have two parts to them. The first part, the primary AI, is the black-box AI. The second part, the secondary AI, generates a post-hoc representor. For explainable AI, the representor is material from which an explanation for the output of the primary AI can be constructed. This material can come in different forms. For instance, it could be a local linear equation that emulates the behaviour of the black-box in a range of nearby cases. Consider, for example, LIME (Ribeiro et al., 2016), an explainable AI model which generates a representor which is locally interpretable. This means that the output of the secondary AI is an equation which tries to model, with a high degree of fidelity, how the primary AI actually works in the given case and nearby similar cases. Other types of representors could involve a representative case that aids in case-based reasoning (Nugent & Cunningham, 2005; Li et al., 2017; Weber et al., 2019). Here, the current treatment recommendation is explained by reference to a sufficiently similar case. The parameters that contributed to the recommendation are used to sort the training data into groups. Of these, one group will adequately represent the parameters that determined the outcome. A representative member of this group will be presented as a comparison to the case which the AI is generating a recommendation for.

Not all post-hoc models count as explainable AI by our lights. In this paper, we reserve the term explainable AI for *high-fidelity* post-hoc models.

*High-fidelity*: A high-fidelity post-hoc model is one in which the representor generated by the secondary AI emulates, to a high degree, the way the primary actually arrived at its result.

Basically, high-fidelity models manage to achieve local transparency. Local transparency provides users with information about how the primary algorithm arrived at a particular decision. This information, by contrast with white-box models, is valid or accurate only under circumstances sufficiently similar to the primary case which was “explained”. Where the parameters differ significantly, entirely different considerations might be salient. Explainability, in this sense, is a weaker specification of the transparency desideratum. Instead of providing information about how all decisions are made, explainable AI only provides information about how *that* decision was made.

This way of specifying explainable AI may be narrower than others have done so. For instance, on other ways of specifying explainable AI all post-hoc models might count as explainable AI (Escalante et al., 2018; Babic et al., 2021; Rudin, 2019). Correspondingly, one criticism levelled against post-hoc models is that their claim to high-fidelity is spurious (Babic et al., 2021; Rudin, 2019). Quite rightly, they point out that it is unclear if such post-hoc methods are actually able to open up the black-box. Our reconceptualization of explainable AI partly immunises it from this criticism. Any AI which does not accurately tell us how its decision was arrived at is not explainable. Of course, it then becomes an open question whether any given post-hoc model is genuinely explainable, but that is a separate question. Insofar as we can be reasonably sure that a given post-hoc model succeeds in allowing us to genuinely trace back how the output depended on the input, it is explainable. This allows us to focus on the value question: Is explainability in AI enough? What about when coupled with reliability? The answer that our paper gives to both questions is in the negative: At the very least algorithmic justifiability is needed as well!

### Justifiable AI

By contrast with the demands of transparency, justifiability in our context pertains to answering two questions: Firstly, there is the question of “is this decision correct?”. That is, does the recommendation fit the patient’s values? Secondly, there is a question of “what reasons do we have for thinking that this decision is correct^7^?” To contrast what algorithmic justifiability requires with what algorithmic transparency requires, we introduce a hypothetical model we call Justifiable AI. While Justifiable AI is also a post-hoc model, it differs from explainable AI in that the justification generated by the secondary AI needs to also make explicit which value commitments would justify the primary AI’s decision. In this way, it is similar to value-flexible AI in that it aims at accounting for the value pluralism found in society.

However, unlike value-flexible AI, there is no need to gather every possible nuance of the patient’s values. Instead, only the broad outlines of the patient’s values and priorities need to be gathered. Since the justification generated is able to specify what value commitments would justify the primary AI’s decision, we end up with two possibilities. Where the primary AI’s decision happens to fit the patient’s values, the justification makes it clear why it is able to do this. In this case, the particular nuances of the patient’s values were irrelevant. On the other hand, if the decision does not fit the patient’s values, the justification should be able to account for why it did not fit. A sufficiently detailed chain of reasoning would make clear which assumption or inference was mistaken. The patient, who is still in-the-loop, would be able to then, with a better understanding of the choices facing her, make a decision. This allows the overall decision process to be more completely value-flexible without necessarily incorporating full value-flexibility into the justifiable AI itself. In this way the goals of full value-flexibility can be achieved while avoiding the infeasibility of fully value-flexible black-box AI.

As mentioned earlier it is possible to incorporate some amount of value flexibility into justifiable AI, but not so much that using the AI becomes onerous. To see how this can be, consider, for instance, Meier et al’s (2022) model. They present a hybrid model of medical ethics decision making where the weights given to various principles can be determined either by the AI algorithm or modified by the user. Given that this model exhibits some degree of value-flexibility, it can be made justifiable by the addition of an appropriate secondary-AI to this system. The secondary AI would generate a low-fidelity representor similar to that devised by Akata et al., (2018) except that the patient’s moral values and preferences are also among the parameters that account for the primary AI’s decision.

Akata et al., have devised a low-fidelity representor for a primary AI which is able to classify images of birds. The primary AI is able to sort birds into various classes where each class represents a species. The secondary AI is, at base, a natural language model. It takes theoretical information about the class and tries to match this to an image-specific description. This is achieved by using an AI model (reinforcement learning). This attempt to harmonise class-specific and image-specific information results in a natural language justification for why the bird in the image belongs to a particular species. Notably, this is a low-fidelity representor because there is no reason to think that the particular features cited by the secondary AI as reasons to think that a given bird is actually, for instance, a Western Grebe were the features that the primary AI used to determine that it is a Western Grebe.

In the medical case, instead of image-specific information, we would have the clinical signs, symptoms, patient history and even some rough information about patient’s preferences. Our classes would be courses of treatment and the class-specific information would be theoretical information about the costs and probable effects of those courses of treatment. These set the parameters for what is choice worthy for a patient. If a given course of treatment would extend life by two months but impose a certain level of discomfort, this sets boundaries on what the patient’s values would have to be like in order for that course of treatment to be choice-worthy for her. Here, the secondary AI would extrapolate from the theoretical features and considerations that make a given treatment recommendation choice-worthy to the specifics of the case in order generate one or more putative justifications for the primary AI’s recommendation. These putative justifications aim at relating the patient’s values to the recommendations provided by the Primary AI. While we grant that medical decision making can be significantly more complex than ornithological classification, it is plausible that something building on Akata et al’s and Meier et al’s models can fulfil this task. Recent advances (Porsdam Mann et al., 2023) in Large Language Models (LLMs) also make the prospects of such an AI more promising. This may especially be the case if the secondary AI can generate multiple possible justifications.

Here is how Justifiable AI is supposed to work: We can re-examine the Chemo case. Alice wishes to attend her granddaughter’s birth in 6 months’ time. Suppose that a Justifiable AI is assessing whether Alice is suitable for chemotherapy. As in the Chemo case, the primary AI says that she is not since it does not know that she has such a preference. However, unlike Chemo, there is a secondary AI, based on an LLM, which churns out one or more plausible justifications. In this case, there is only one, namely, that chemotherapy is not optimal because the therapy can only extend her life by 6 months at the cost of moderate decreases to Alice’s quality of life. The physician, on his part, can verify that the justification given both fits the medical facts of the case and is plausible given existing medical knowledge. Alice is now able to point out to the physician that she wishes only to attend her granddaughter’s birth and is willing to bear with the moderate discomfort in order to do so. After becoming aware of the secondary AI’s justification, the physician should be able to see that this justification is defeated by the considerations Alice raises. As such, they ignore the decision made by the primary AI and Alice decides to go on chemotherapy.

To generalise, the justifications provided by the secondary AI provide a starting point for the physician and patient to jointly deliberate about the latter’s options. The justifications do this by providing plausible lines of argument that would justify the primary AI’s recommendation. These arguments would make clear what would need to be assumed in order for the recommendation to be justified. The physician’s role is to verify the cogency of these justifications and ensure that they fit both the facts of the case and established medical knowledge. This allows the patient to evaluate these assumptions and check whether her values and priorities match or defeat these assumptions. It may turn out that one or more of these putative justifications fit the patient’s values or at least can be easily modified to fit the patient’s values. If so, the patient can accept the primary AI’s recommendation. Otherwise, if none of the justifications can be made to fit the patient’s values, she will reject the recommendation.

### Wrong information

We are now in a position to see how interpretable and explainable algorithms provide the wrong kind of information. Knowing how the primary algorithm actually made its decision is neither necessary nor necessarily sufficient to determine whether to accept the recommendation of the primary algorithm. To illustrate, suppose that a patient, Hal, was looking at a LIME system AI and can see why given the information provided, palliative care instead of chemotherapy was recommended by the AI. However, this explanation invokes considerations that plausibly have little relation to whether he should go for chemotherapy. For instance, suppose that the primary algorithm heavily weighted the fact that Hal likes to play badminton in how it actually came to the recommendation. This could arise simply because in the training data, for some reason or other, being a badminton player was strongly correlated with the judgment that palliative care maximised QALYs. An explanation such as that given by the LIME system would make explicit that the primary algorithm used the fact that Hal played badminton as a proxy to determining that palliative care maximised QALYs. However, it would not be clear whether playing badminton is a good proxy for some ground truth about factors that affect QALY or whether it reflects some sort of bias in the training data. It is possible that researchers might systematically over-value the ability to play one’s preferred sport in assessing quality of life. Unknown to Hal, this decision to recommend palliative care is actually justifiable to him. For instance, the longevity-discomfort trade-off is such that even if Hal was not a badminton player, palliative care is better for him than chemotherapy. However, by being provided the explanation, but not the justification, Hal is in no better epistemic position to determine whether to accept or reject the recommendation. In fact, Hal might be in worse position as he might reject the recommendation on the basis that a machine reached it on the basis of what seems like an irrelevant consideration. As a result, he ends up taking a course of treatment (chemotherapy) which makes him worse off.

A Justifiable AI here would have given him reasons for palliative care which are not necessarily faithful to how the decision by the primary AI was actually computed. That is, by contrast with LIME, it would generate a representor which need not be faithful to how the decision was made by the primary algorithm. A Justifiable AI would aim to provide a chain of reasoning for its recommendations that accounts for all morally important considerations regardless of whether these accurately model how the primary AI arrived at this decision.

Suppose, instead, that there was a second secondary AI which did provide a chain of reasoning for the primary AI’s recommendation and this managed to show Hal that palliative care did fit his values. For instance, it could say that it recommends palliative care because the side effects of chemotherapy are tiredness, nausea, mouth sores and reduced appetite and few people are willing to tolerate such effects for only a six-month extension of life. Hal, meanwhile, might consider himself a foodie and agree that he would not like to spend his last days like that. Then even if Hal knew, via the first secondary AI, that the primary algorithm arrived at its recommendation by reference to an irrelevant consideration, Hal would have little reason to reject the AI’s recommendations as the second secondary AI would show why the recommendation was justifiable to him. If this is right, then Justifiable AI is required to ensure that decisions made, especially in cases where it is not immediately obvious which decision is justified, are justifiable to patients.

One might, nevertheless, worry that patients would reject the recommendation upon learning that the algorithm made said recommendation on the basis of bad or irrelevant reasons because they feel alienated from the recommendation. As such, or so the objection goes, algorithmic transparency is important so that we ensure that patients have no grounds for being alienated from the decision.

However, it is not clear if patients would in fact reject recommendations that happened to be made by the algorithm for bad reasons despite knowing that there are good reasons in favour of them. After all, we know that some people support the abolition of slavery for bad reasons. A Kantian and a utilitarian may each regard the other’s reasons for abolition as objectionable or irrelevant. However, the mere fact that we know that such people exist does not undermine our condemnation of slavery and our support for its abolition. It is unclear, then, why patients would be alienated by a mere machine reaching, on the basis of bad reasons, a decision which they have good reason to endorse anyway.

Nevertheless, even if patients are inclined to reject a recommendation due to being alienated from the grounds on which it was actually made, doing so would not be rational. After all, the actual decision-makers, namely the physician and the patient, if they were to decide on the basis of the reasons they know justify the recommendation, would have arrived at the decision on the basis of good reasons. The irrationality of rejecting justified recommendations would count against algorithmic transparency. While it can be objectionably paternalistic to withhold information that is relevant to the decision, it is not paternalistic to withhold irrelevant information which is potentially distracting and alienating.

The key point to note here is that most of the time, explainable and interpretable AI would not be able to provide information that is relevant to justifying the decision. By contrast, justifiable AI, if it works as intended, will provide information^8^ that is needed for justification.

## Conclusion

Summing up, we have argued that one normative constraint on medical decisions is that they fit patient values and that the patient understands why they do. This is grounded in what is now accepted as best practice: shared decision-making in the clinical setting, and indirectly, by considerations of beneficence, autonomy and respect. We have then argued that this requires algorithmic justifiability and that algorithmic transparency is insufficient because it is neither necessary nor necessarily sufficient for justifiability. If all this is right, then AI researchers should be working to implement algorithmic justifiability. Likewise, national guidelines on AI should at least be requiring justifiability. This suggests a further question of whether justifiability should fully supplant or merely complement transparency as a requirement of medical AIs. However, there is not space in this paper to engage properly with this further question, and so we reserve it for future work on justifiable AI.

## Data Availability

Not applicable.

## Footnotes

1 Elgin (2017) makes a similar point with respect to scientific theories and truth, namely that such theories need not align perfectly or even very much with the truth to be successful or correct.

2 See also Meier et al., (2022) for a recent attempt to use AI in ethical decision making in the clinical setting.

3 Such standards could be anything like truth, moral permissibility, scientific validity, counterfactual robustness or being adequately supported by the available evidence etc.

4 The distinction between the algorithm actually weighing considerations in the right way and the decisions fitting those considerations is analogous to the distinction between doxastic and propositional justification in epistemology. The former is concerned with whether a given token belief is formed in the right way (Turri, 2010) or whether it was based on the evidence (Swain, 1979; Pollock & Cruz, 1999; Korcz, 2000). The latter is concerned with whether a belief is supported by the evidence or whether it is possible for the agent have such a belief and it be doxastically justified (Turri, 2010).

5 Interpretability is sometimes cashed out in terms of an outcome being understandable to persons as opposed to merely knowing how a model will respond to any set of inputs. Our use of the term is in part stipulative. In addition, those who do have the requisite background knowledge in computer science and the domain in question are, in principle able to make sense of these fully transparent algorithms. Under these assumptions, the two senses of the term interpretable dovetail (Broniatowski, 2021). It should be noted that even here, understanding how the algorithm made its decision oes not suffice for understanding why the decision fits the patient’s values.

6 Some like Wachter et al., (2018) dispute that explainability is about transparency at all. However, despite initially seeming to aim at something else, her account of explainability still involves tracing the path-dependence of the algorithm’s outputs on its inputs (Durán, 2021). It thus involves at least partial transparency. More importantly, the aim of this paper is to explore a distinction between explainability and justifiability that has been hitherto overlooked. It should come as no surprise that some authors will conflate the two.

7 There might be worries that talk about correctness is too metaphysically laden. To understand what we mean by ‘correct,’ a decision is correct if and only if it meets all the relevant normative standards. In the clinical context, one of those standards is that the decision adequately fits the patient’s values and concerns. This understanding of correctness is compatible with realist, constructivist, conventionalist and even fictionalist accounts of normative standards. Given the ecumenicism of this account of correctness, such worries are misplaced. There is also, it seems, a perfectly ordinary sense in which decisions which were morally or prudentially right are called correct while those which are morally or prudentially wrong are called mistakes. This suggests a deflationary account of decisional correctness: “Palliative care is correct for David just in case it fits his priorities and…”.

8 One might argue that justifiable AI does not really provide new information, but merely reminds people of what they already knew and connects that to appropriate recommendations, but we need not concern ourselves with such subtleties in this paper. However we describe what justifiable AI provides, it aids the patient in making decisions that are justifiable to her.
